# Differences between two sequential uncemented stem sizes in total hip arthroplasty: A comparative biomechanical study and potential clinical implications

**DOI:** 10.1051/sicotj/2022043

**Published:** 2022-11-11

**Authors:** Katherine Wang, Eustathios Kenanidis, Khurram Suleman, Mark Miodownik, Mahsa Avadi, David Horne, Jonathan Thompson, Eleftherios Tsiridis, Mehran Moazen

**Affiliations:** 1 Department of Mechanical Engineering, University College London Torrington Place London WC1E 7JE UK; 2 Academic Orthopaedic Department, Papageorgiou General Hospital & CORE Lab at CIRI AUTH, Aristotle University Medical School, University Campus 54 124 Thessaloniki Greece; 3 DePuy Synthes St. Anthony’s Road Leeds LS11 8DT UK

**Keywords:** Total hip arthroplasty (THA), Biomechanics, Uncemented, Experimental, Stiffness, Strain, Stem size

## Abstract

*Background*: Early failure of uncemented femoral stems associated with incorrect sizing is a known postoperative complication. Surgeons are often faced with the question of whether an uncemented stem of adequate stability or a larger-sized stem should be implanted, especially when the proximal femoral cancellous bone is adequate. The biomechanical effect of sub-optimal stem sizing in the femur remains unclear. This study investigated the mechanical behaviour of two sequential sized uncemented stems of the same type. *Methods*: Six laboratory models of synthetic non-osteoporotic femora were randomly divided into two groups and implanted with either a nominal or oversized uncemented hydroxyapatite-coated nonporous titanium collarless stem. Stiffness, uniaxial strain, and pattern of strain distribution were measured under an anatomical one-legged stance. *Results*: Oversized stems demonstrated a higher overall stiffness compared to nominal; however, this was not statistically significant. The nominal stem showed a higher strain in the neck and the proximal medial diaphyseal region. The oversized stem showed higher strains in the distal region around the implant tip. *Conclusion*: Opting to use a larger stem may potentially increase primary stability, thus allowing safer early mobility. However, higher stiffness may lead to stress shielding, bone loss, and thigh pain in the long term. In addition, strains in the diaphysis and the tip of the stem may predispose to periprosthetic fractures, especially in osteoporotic bones, making this a relatable aspect for users and biomechanical loading. Given the wide range of complex factors that need to be considered when choosing stem size in uncemented THA surgery, this study’s results should be interpreted cautiously.

## Introduction

Early failure of uncemented femoral stems associated with incorrect sizing in the host femur has been documented [1, 2]. Loosening may be patient, implant or surgeon-related, for example, femoral anatomy, stem design, and surgical approach. The incorrect entry point may lead to varus position and stem under-sizing. Subjective evaluation or damage of the metaphyseal cancellous bone during stem insertion may also be implicated [3, 4].

The surgeon’s choice of the correct stem size remains unclear. At the end of the femoral preparation, the surgeon’s decision for the stem to be implanted is subjective. Surgeons are often faced with whether a larger or nominal-sized stem should be used, especially when the proximal femoral cancellous bone is adequately compacted [3, 4]. The good quality compacted cancellous bone should not be scraped off the metaphysis, and unfortunately, preoperative templating and/or intraoperative radiographs cannot assess its suitability and hardness.

To the best of our knowledge, no biomechanical study has made a direct comparison between the performances of two sequential sizes of the same femoral stem design. Among the two sequential stems, the first one was selected for the experiment according to surgeon templating (nominal), where the nominal stem was considered stable, and the second was one size up and forced to be implanted into the same randomised femur (oversized). This study aimed to investigate the biomechanical effect of two sequential-sized uncemented stems of the same type in total hip arthroplasty and whether using an oversized stem would produce a different biomechanical effect in the same femora.

## Materials and methods

### Specimens

**Figure 1:**
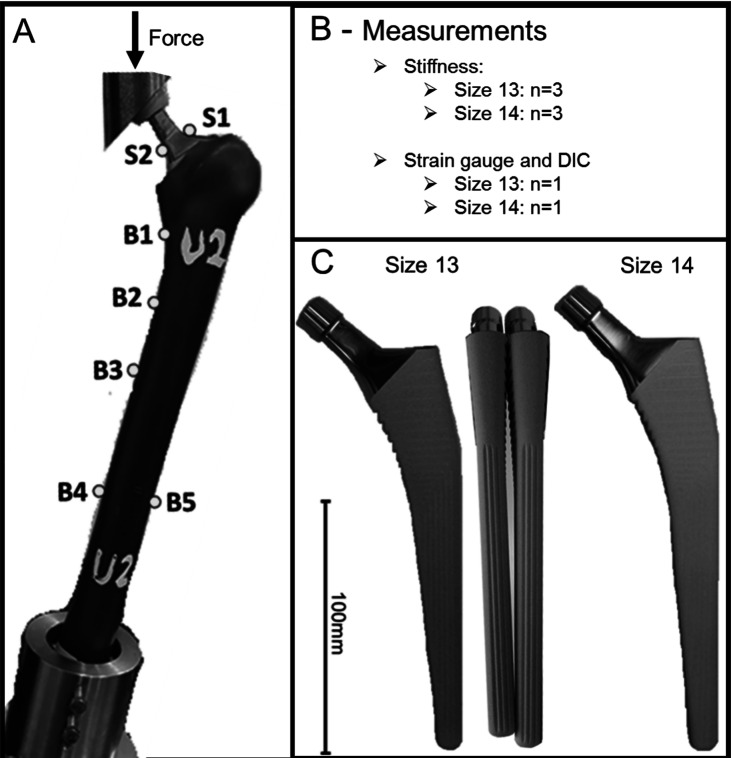
Overview of the study. Experimental setup of total hip replacement (A), measurements obtained (B), and the two different stem sizes used (C). S1–S2 highlight the strain gauge attachment site on the stem, and B1–B5 highlight the strain gauge attachment site on the bone. Size 13 *N* = 3.

This study used six large left, fourth-generation composite femurs (Sawbones Worldwide, WA). The femora were randomised into two groups with three constructs in the size 13 group and three in size 14 group to simulate uncemented THAs (Fig. 1). The stem sizes were determined by an orthopaedic surgeon as the final stem to be implanted. The surgeon performed uncemented THA according to the manufacturer’s surgical technique in the first three Sawbones, selecting stem size 13 (nominal) as the stem to be implanted according to templating. It should be noted that: (a) the nominal stem was deemed appropriate stability according to surgeon experience. The surgeon then repeated the experiment in the other three femora broaching further to allow a stem size 14 (oversized) as the one sized up uncemented Corail stem; (b) Synthetic bones were used as opposed to cadaveric bones to minimise the variability of the bone quality and its potential impact on the outcomes of this biomechanical study; (c) Single size synthetic bones were used in this study to purely investigate the difference between two sequentially sized stems.

Specimens were prepared by removing the femoral condyles, 60 mm distal of the femur. Uncemented THA was performed using a DePuy Corail, standard offset, collarless (DePuy Synthes, Leeds, UK) in size 13 and size 14 and an Articul/Eze femoral head (28 mm diameter). This stem fully hydroxyapatite-coated nonporous forged titanium stem with a tapered wedge design and a trapezoid cross-section proximally and quadrangular cross-section distally metaphyseal loading [1]. The femoral canal was broached to the appropriate size, and the stem was inserted by press-fit. The implant had a young’s modulus of around 110 GPa. Note the broach was the same size as the corresponding stem without the hydroxyapatite coating.

### Loading

The distal 40 mm section of the femur was fixed securely in a cylindrical housing using screws and mounted on a material testing machine (Instron, Massachusetts, USA) at 11° adduction in the frontal plane and aligned vertically in the sagittal plane to simulate an anatomic one-legged stance (e.g. [6]). Constructs were tested under displacement control at a rate of 2 mm/min to a maximum of 500 N, corresponding to the recommended partial weight-bearing (e.g. [7]). Loading was applied to the head of the femoral stem via a hemispherical cup [7–10]. Each construct was tested six times to obtain the average measurement.

### Measurements and analysis

All six constructs were tested to obtain the overall stiffness of each specimen. Stiffness was calculated from the slope of the load-displacement data obtained from the materials testing machine. The specimen with the value closest to the overall average stiffness from each group was then used to measure surface strain (Fig. 1).

*Strain Gauges:* Uniaxial strain gauges were attached at seven sites on the construct. All gauges on the bone were positioned so that the axes were aligned with the femur’s longitudinal axis. Gauges on the stem were aligned with the longitudinal axis of the stem neck. Two gauges with a gauge length of 0.2 mm (FLGB-02-17, Tokyo Sokki Kenkyujo, Tokyo, Japan) were placed on the medial and lateral side of the stem neck (S1–S2), five gauges with a gauge length of 3 mm (GFLAB-3-50, Tokyo Sokki Kenkyujo, Tokyo, Japan) were attached to the surface of the femur. Four gauges were positioned on the medial length of the femur at 0, 40, 80, and 200 mm distal to the lesser trochanter, with an additional gauge positioned on the lateral side of the femur, 200 mm distal to the lesser trochanter (B1–B5) (Fig. 1). Values in positive denote strain in tension, and values in negative denote strain in compression.

*Digital Image Correlation (DIC)*: A DIC system consisting of a pair of two high-resolution cameras were used to create a stereo view of the medial femoral surface, from which the surface strains were calculated using the DIC programme Vic-3D 8 (Correlated Solutions Inc, Irmo, SC, USA). The cameras were mounted on a rigid beam, which was mounted on a floor-standing tripod. DIC stereo image pairs were recorded from the two cameras and processed using the VIC-3D 8 software. The surface distributions of the maximum principle strains were analysed. A white-on-black speckle pattern using high-contrast spray paint was created on the bone’s medial side for the THA specimens (e.g. [11, 12]).

*Statistical analysis:* Two-tailed, unpaired Student t-test at a level of significance of *p* < 0.05 was used to detect significant differences in the stiffness and strain. The strain measurements were performed only on one specimen in each group; hence the *p*-value is based on the results from the same specimen that was repeated (loaded and unloaded in the material testing machine) six times. Stiffness measurements were carried out in all six sawbone constructs.

## Results

### Axial stiffness

**Figure 2:**
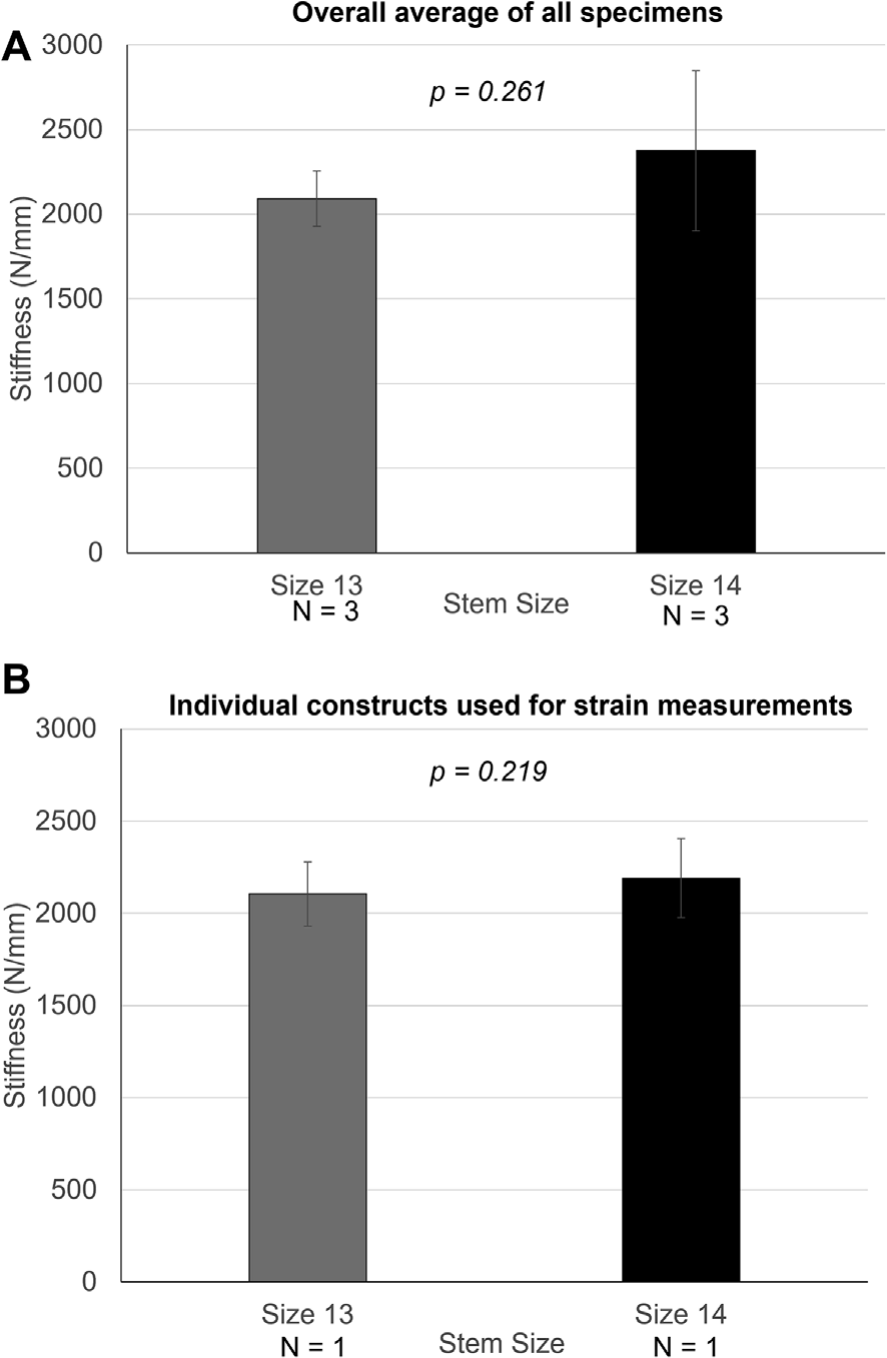
(A) Comparison of overall average stiffness between size 13 and 14 uncemented stem specimens based on the overall average. (B) Comparison of average stiffness between individual constructs of size 13 and size 14 stems. Asterisk (*) denotes the statistical difference between the two variables (*P* < 0.05).

A comparison of the overall average stiffness between the two groups is shown in Figure 2A. Constructs with oversized stems (size 14) were stiffer under axial compression compared to the constructs with nominal stems (size 13). However, this was not statistically significant (*p* = 0.26). When considering the individual constructs’ stiffness closest to the overall average, the larger stem size also showed higher stiffness than the nominal stem, albeit with no statistical difference (Fig. 2B).

### Strain

**Figure 3:**
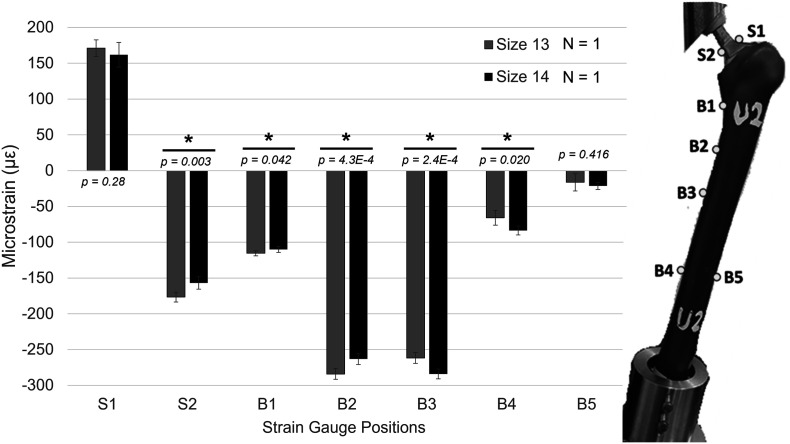
Summary of the strain measurements taken from strain gauges on different locations of the construct under a 500 N axial load. Uncemented size 13 and size 14 hip stems compared. *Highlights the statistical difference between corresponding groups (*P* < 0.05).

**Figure 4:**
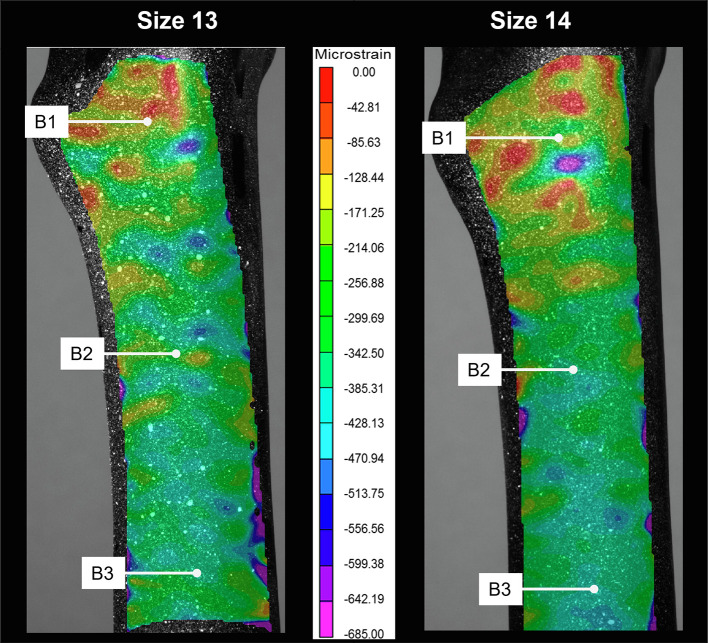
Comparison between the pattern of maximum principal strain across the medial side of the bone, between the size 13 and size 14 group at 500 N axial load.

A comparison of the average strain between the two constructs is shown in Figure 3. Strain gauge results showed that strain on the stem neck was higher in size 13 than size 14 at S2 (Fig. 3). The highest strain for both groups was at the medial side of the mid-shaft (B2 and B3). The size 13 construct had higher strain than the size 14 construct at B1 and B2 (*p* < 0.05), i.e., in the femur’s proximal medial section. This pattern was reversed at locations in the mid and distal regions of the bone, where the size 14 construct had higher strain compared to the size 13; see Figure 3 for B3–B4 (*p* < 0.05) and B5 (*p* = 0.416). Strain decreased distally along the bone. The difference in stem length could contribute to the strain change observed in B3. DIC results (Fig. 4) showed that the overall pattern of the third principle strain (maximum compressive strain) on the bone’s medial side was lower (in terms of absolute magnitude) in size 14 construct compared to the size 13 construct. This pattern was in line with the strain gauge data.

## Discussion

This study investigated the biomechanical effect of two sequential in size, uncemented tapered collarless stems in an experimental setting and how they may perform under an anatomically representative one-legged stance. The nominal-sized stem was considered of appropriate stability, however, the surgeon felt that a larger size could also be used. Both stems might be the surgeon’s final choice at the end of the femoral preparation, thus providing insight into how they may perform in a clinical setting. This study aimed to shed some light on the potential biomechanical impacts of this decision-making process.

This study used two methods, strain gauge and DIC, to characterise the mechanical strain across the constructs. In summary, our results indicated that stem size 13 appeared to have a higher strain on the neck and proximal medial metaphyseal region, and stem size 14 had higher overall stiffness and higher strains in the lower medial femoral diaphysis and the tip of the stem. However, it should be noted that there was no statistical difference between the stiffness of the two different stem sizes.

The higher stiffness of stem size 14 may potentially lead to a lower risk of subsidence under loading. Consequently, it may allow a faster full weight-bearing during the immediate postoperative period. Size 13 stem showed lower overall stiffness and higher strain in the neck and the medial metaphyseal femur than stem size 14. This observation may explain a potential higher varus loading of a nominal stem and subsequent higher risk of early loosening of the prosthesis. It is noteworthy to comment on the offset of the stem neck; size 14 has a higher offset (42.3 mm) compared to size 13 (41.7 mm). The offset of the stem may alter the moment of the arm, which could potentially explain the differences in strain found in the stem neck. However, Rod Davey et al. [13] suggested that the lever arm of the bending moment increases because of an increased offset, whereas the bending moment only marginally increases due to a decrease in the resultant force. Hence, the net change in strain in the medial cortex is thought to be small.

In both constructs, the highest strains were found in the mid-diaphysis’s medial side; this area of strain concentration can be explained by the modulus of elasticity mismatch between the distal section of the femoral stem and the bone. This discrepancy could cause high stress in the femoral diaphysis when weight-bearing loads are transferred from the stem to the femur. From a clinical perspective, the high strains observed in B2 and B3 sensors (Fig. 1) are where Vancouver type B fractures may occur [14–18].

The oversized construct had higher overall stiffness and increased strains on the medial diaphysis and tip region compared to the nominal size. The latter observations potentially may have a dual clinical implication; first potential higher proximal stress shielding and, second, a higher risk for a Vancouver type B PFF [19, 20]. In a more refined data analysis, size 14 (oversized) is slightly longer than 13 (nominal). The difference in stem length could explain the strain change in the B3 sensor. Higher overall stiffness and strain at the tip of the stem in the larger 14 stem supports the theory of higher stress shielding and/or increased PFF risk shown in previous studies [21–23]. However, given that no statistical difference in stiffness between the two sizes was observed in this study, caution must be taken when interpreting this data. A recent computational finite element study that analysed the effect of four different collared stem sizes of the same design: oversized, ideal (nominal) sized, and two sizes down stems; concluded that sizing and positioning might impact primary stability but is unlikely to affect bone resorption [24].

Choosing the correct femoral stem size is crucial in determining the initial stability, osseointegration, and optimal stress distribution in the proximal femur. This study examined the cortical strains in uncemented press-fit stem implantation into the synthetic bone immediately postoperative scenario. Our analysis indicates what might occur immediately postoperatively when osseointegration has not yet occurred. In addition, the results may not be reproducible for other stem designs. Engh et al. [21] found no statistical relationship between the size of the uncemented extensively porous-coated stem of certain design and revision, loosening, pain, or patient satisfaction; despite the demographic differences between the groups, they found an overall consistency in clinical outcome. However, given the wide range of complex factors that need to be taken into account when choosing stem size in uncemented THA, results from this study should be interpreted with caution, given that surgical procedure is often based and clinical experience, which differs between hospitals and regions of the world [21, 23, 25].

In uncemented THA, the surgeon is subjectively evaluating two options when dealing with the metaphyseal cancellous bone. First, reaming up to almost the cortical bone to achieve primary stability against it or retaining, rasping and compacting the cancellous bone creating a neo-endosteum that can accommodate the stem and subsequently achieve the expected osseointegration [3]. While these two philosophies exist, it is generally well understood that certain implant designs are intended for use with one or another philosophy. The Corail stem is intended to preserve a cancellous bone bed; thus, the surgical technique should be referenced to indicate this. Conclusions drawn on bone-retaining philosophy stems (such as the Corail) should not be extended to all uncemented stems.

There are several limitations to this study: (1) evidently, we cannot evaluate the long-term bone-in and on-growth in the bone-implant interface and how this would affect biomechanics. (2) The effects of muscle forces were not included. (3) One of the size 14 specimens showed a much higher stiffness than the other two (Table 1) and could be considered an outlier. Nonetheless, the specimen from this group that was the closest to the overall average stiffness used to obtain results from the strain gauge data showed a statistical difference, suggesting that the results obtained are still valid. (4) The sample size for each group was small. (5) While Sawbones are a reasonable surrogate, they do not completely represent bone.

**Table 1:** The average stiffness and standard deviation for each specimen is shown. Each specimen underwent six repeat tests. The asterisk (*) indicates the specimen used for strain testing.

	Size 13			Size 14			Comparison of size 13 and 14
Specimen	U2*	U3	U4	U5*	U6	U7	*P*-Value
Average	2105	1977	2192	2191	2093	2843	0.26
SD	174.0	95.7	131.4	215.0	92.5	534.3	

X-ray radiographs were analysed, and it determined that all stems were consistently aligned in the Sawbone, however, future investigations should consider the analysis of the cavity produced during broaching to determine if differences in implantation method (e.g., varus alignment or particular contacting points in the cortex).

Considering the aforementioned limitations and several others discussed throughout the study, it is likely that the absolute values reported in this study would be different from the *in vivo* data; nonetheless, we believe that the relative differences considered in this study remain valid and shed light on the possible biomechanical impacts of commonly seen decision-making process in surgery.

In conclusion, this study highlights the possible biomechanical differences associated with implant selection and the potential impacts of this decision-making process, i.e., between two sequential-sized uncemented collarless stems that could be used in the same procedure. The results found that the oversized stem showed higher stiffness and strains close to the stem tip. The nominal stem demonstrated higher strains in the stem neck and proximal medial diaphysis. The former may indicate better stability, but potentially a higher risk of a Vancouver type B fracture or proximal stress shielding, and the latter may indicate a higher risk of loosening following mobilisation. However, given the complex factors that need to be considered, results from this study should be interpreted with caution. This preliminary study merits further investigation in a more rigorous study with larger numbers where the effect of collared vs. collarless implants can also be investigated.

## Conflict of interest

The authors declare no conflict of interest.

## Funding

This work was supported by DePuy Synthes (Leeds, UK) and EPSRC Doctoral Training Partnership (DTP) Case Studentship (539270/173067).

## Ethical approval

Ethical approval was not required.

## Informed consent

This article does not contain any studies involving human subjects.

## Author contributions

KW (First author): performed investigation and acquisition of data, formal analysis, interpretation of data, writing – original draft, review and editing. MM (Corresponding author): conceptualization, formal analysis, supervision, methodology, project administration, writing – review and editing. EK, KS: performed laboratory work and investigation, writing – review and editing. MA, DH, JT: conceptualization, resources, supervision, writing – review and editing. MM: conceptualization, supervision, writing – review and editing. ET: conceptualization, supervision, methodology, writing – review and editing.
